# Verification of Chromatographic Profile of Primary Essential Oil of *Pinus sylvestris* L. Combined with Chemometric Analysis

**DOI:** 10.3390/molecules25132973

**Published:** 2020-06-28

**Authors:** Martina Allenspach, Claudia Valder, Daniela Flamm, Francesca Grisoni, Christian Steuer

**Affiliations:** 1 Institute of Pharmaceutical Sciences, ETH Zürich, HCI, Vladimir-Prelog-Weg 4, 8093 Zürich, Switzerland; martina.allenspach@pharma.ethz.ch (M.A.); francesca.grisoni@pharma.ethz.ch (F.G.); 2 Systema Natura GmbH, Konrad-Zuse-Ring 8, 24220 Flintbek, Germany; science@systemanatura.de (C.V.); science1@systemanatura.de (D.F.)

**Keywords:** *Pinus sylvestris* L., primary essential oil, chromatographic profile, chemometric analysis

## Abstract

Chromatographic profiles of primary essential oils (EO) deliver valuable authentic information about composition and compound pattern. Primary EOs obtained from *Pinus sylvestris* L. (PS) from different global origins were analyzed using gas chromatography coupled to a flame ionization detector (GC-FID) and identified by GC hyphenated to mass spectrometer (GC-MS). A primary EO of PS was characterized by a distinct sesquiterpene pattern followed by a diterpene profile containing diterpenoids of the labdane, pimarane or abietane type. Based on their sesquiterpene compound patterns, primary EOs of PS were separated into their geographical origin using component analysis. Furthermore, differentiation of closely related pine EOs by partial least square discriminant analysis proved the existence of a primary EO of PS. The developed and validated PLS-DA model is suitable as a screening tool to assess the correct chemotaxonomic identification of a primary pine EOs as it classified all pine EOs correctly.

## 1. Introduction

Pine trees are evergreen conifers belonging to the genus *Pinus* of the family *Pinaceae*. The genus *Pinus* exhibits a taxonomical variety of about 115 species and occurs mainly in the northern hemisphere [1,2,3,4]. Needles and twigs are source of pine essential oils (EOs), used to treat respiratory infections based on their antibacterial, anti-inflammatory, expectorant and analgesic potential [5,6,7,8,9,10,11,12,13,14]. EOs are mixtures of natural complex substances mainly categorized into monoterpenes, sesquiterpenes and their oxygenated derivatives and are synthesized as secondary metabolites [15]. Pine EOs consist mainly of monoterpenes and sesquiterpenes [5,7,9,10,11,13,16,17,18]. Additionally, diterpenoids may be present as they are the major compounds of oleoresin, which are accumulated with EOs in resin canals [3,19,20,21,22,23,24,25,26,27,28,29]. Recently, diterpenoids raised the attention of medicinal chemistry as potential fragments and lead compounds in drug discovery [30,31,32].

*Pinus sylvestris* L. (PS) is the most widely distributed pine due to its ability to adapt to various climatic conditions and to grow on different soil types [3,4]. Furthermore, this species shows extensive genetic variability and can mainly be categorized into 3-carene, α-pinene or isoabienol chemotypes [33]. Closely related pine trees are *Pinus cembra* L. (PC), *Pinus mugo* T_URRA_ (PMu) or *Pinus nigra* J. F. A_RNOLD_ (PN) (phylogenetic tree, Figure S1). Due to their morphological similarity, congeners are easily mixed up with PS [5,17,18,34]. The profile of industrial used pine EOs is defined by the European Pharmacopeia (Ph. Eur.): *Pini sylvestris* aetheroleum (PS) and *Pini pumilionis* aetheroleum (PMu) [35]. For quantification, the Ph. Eur. uses a targeted gas chromatography coupled with the flame ionization detector (GC-FID) approach evaluated by on the normalization procedure for 11 compounds. These compounds are α-pinene, camphene, β-pinene, 3-carene, β-myrcene, limonene, β-phellandrene, *p*-cymene, terpinolene, bornyl acetate and β-caryophyllene. The chemical profile of EOs may be influenced by several factors, e.g., geographical and seasonal variations, environmental condition or harvesting period, which may affect the quality [36]. The influences of the different climatic and soil conditions on the secondary metabolome have already been reported on various EOs [37,38]. Thus, for the authentication of herbal products, a powerful approach like chromatographic profiling is highly recommended [39,40]. In recent years, chromatographic profiling is often combined with multivariate analysis to evaluate the relationship between the chemical composition and quality, taxonomic identification or geographical origin [36,41,42]. Hyphenation of the chromatographic profile to multivariate analysis has already been performed in several studies for quality evaluation of EOs [43,44,45,46]. Since standards of primary pine EOs are still lacking and evaluation is challenging, verification of the chromatographic profile of these EOs combined with chemometric methods is needed.

The present work represents a detailed chemical characterization of primary EOs of PS including samples from several geographical origin analyzed by GC-FID and GC hyphenated to mass spectrometer (MS). In contrast to the formerly published literature, we assured the application of a standardized production process on industrial batches and focused on primary EOs only. Moreover, all samples were traceable and derived of significantly different geographical origins to deliver a profound data bases to distinguish characteristic profile differences. Subsequently, we combined the chromatographic profile with multivariate analysis to assess the relationship between the chemical composition, geographical origin or taxonomic identification. The collected data were analyzed using principal component analysis (PCA) and partial least squares discriminant analysis (PLS-DA). Finally, a significant chemical marker for the classification of PS is determined.

## 2. Results and Discussion

### 2.1. Chromatographic Profile

**Figure 1:**
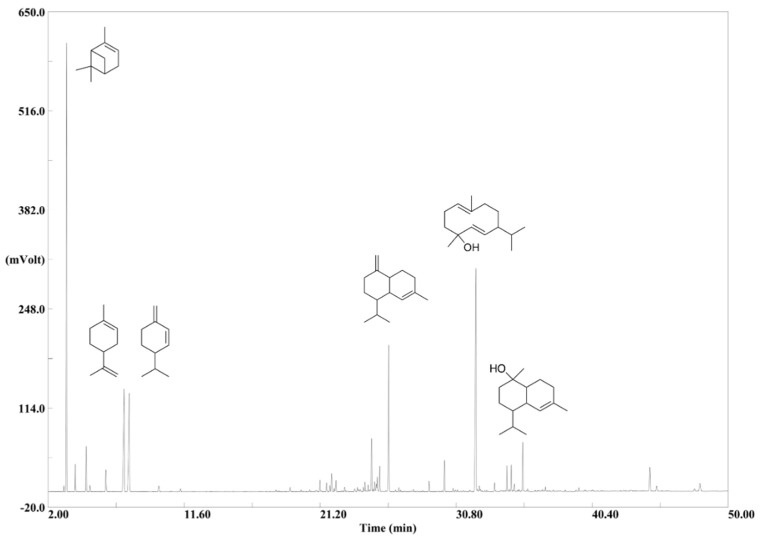
The chromatographic profile obtained by GC-FID of **23** (Russia) with a distinct sesquiterpene profile (20–44 min) and a diterpene area (44–50 min). Larger peaks are exemplified with the molecular structure: α-pinene (3.3 min); limonene (7.3 min); β-phellandrene (7.7 min); γ-cadinene (26.0 min); gemacrene-d-4-ol (32.1 min); α-cadinol (35.5 min).

**Figure 2:**
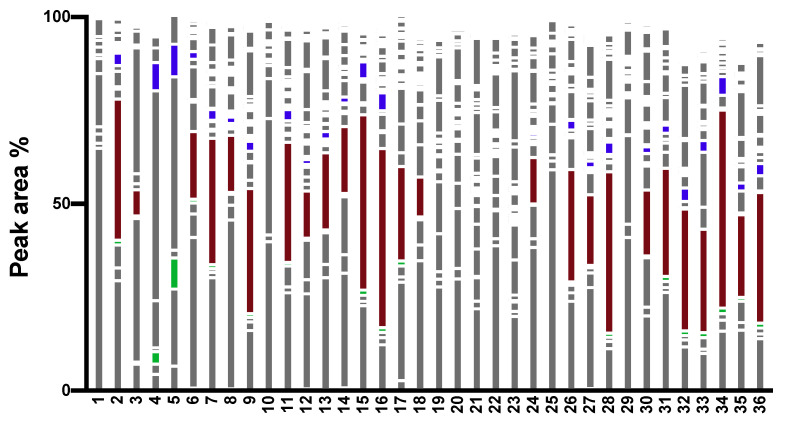
3-carene-rich (red) and 3-carene-low EOs with terpinolene (blue) and sabinene (green).

The chromatographic profile of a primary EO of PS is shown in Figure 1. The chemical composition of the EOs could be classified into monoterpene hydrocarbons, oxygenated monoterpenes, sesquiterpene hydrocarbons, oxygenated sesquiterpenes and oxygenated diterpenes (Table 1, Table S1). The performance of the analytical method was confirmed in an interlaboratory comparison. All analyzed primary EOs of PS (*n* = 36) showed a similar monoterpenoid pattern. As the main compounds, α-pinene (*n* = 19), 3-carene (*n* = 14), β-phellandrene (*n* = 2) or β-pinene (*n* = 1) were verified. The analyzed primary EOs of PS were categorized into 3-carene-rich (>5%) and 3-carene-low EOs, as proposed earlier (Figure 2) [47]. Obviously, terpinolene was detected in 3-carene- and sabinene-rich EOs (**4** and **5**; 3.3% and 8.3%), respectively, which is in line with previous published data [48]. The ratio of 3-carene to terpinolene was found to be 15:1. Interestingly, the monoterpene pattern of the Danish EOs was completely different. Three out of five of the Danish EOs did not contain α-pinene or 3-carene as main compound. The main compounds were either β-pinene or β-phellandrene, respectively.

**Figure 3:**
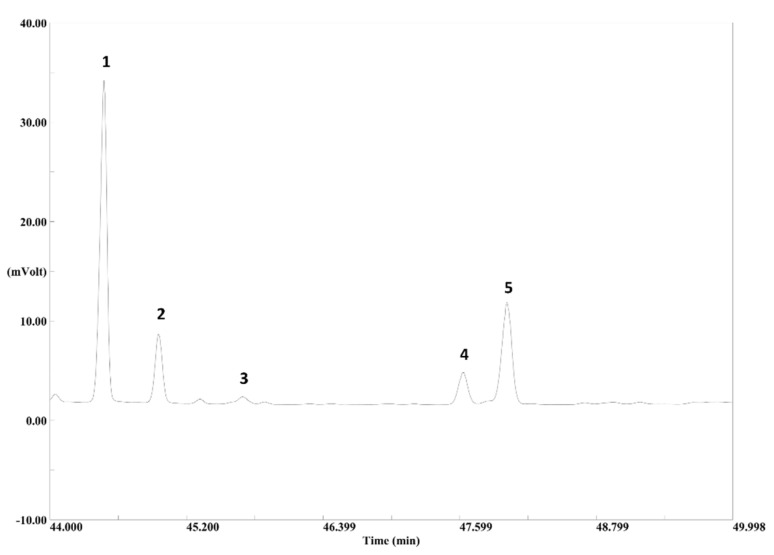
The typical diterpenoid profile of **23** (Russia) obtained by GC-FID: (**1**) isoabienol, (**2**) sandaracopimaral, (**3**) *cis*-abienol, (**4**) palustral and (**5**) isopimaral.

Additionally, the primary EOs of PS contained a typical diterpenoid profile (Figure 3), whose compounds were identified by comparing the mass spectra with the libraries and data in the literature (Figure S2) [22,49,50,51,52,53,54,55]. The identified diterpenoids belong to the labdane, abietane or pimarane group. The diterpenoid profile among the primary EOs was similar, although the abundance of the diterpenoids is also influenced by different environmental factors, genetic conditions and chemical reactivity [33]. The most intense peak of the diterpene area of **1**, **6**, **18**, **21**–**23**, **25**, **26**, **31** and **33**–**36** was identified as isoabienol, which belongs to the abienol group. Isoabienol was mainly found in the needles, whereas it was hardly detected in twigs (Figure S3). The mass spectra of the abienols are characterized by similar fragmentation patterns, which make the identification of the abienols challenging. Nevertheless, the structure of isoabienol was determined by comparing the obtained mass spectrum with the one reported by Adams et al. and was identified by its characteristic base peak at 191 arising from loss of water (H_2_O) with an additional loss of the side chain (C_6_H_9_) [52]. Besides isoabienol, further diterpenoids were present in the primary EOs of PS and were identified as *cis*-abienol from the labdane class, sandaracopimaral and isopimaral from the pimarane class and palustral from the abietane class, all exhibiting high spectral similarity. Interestingly, *cis*-abienol was detected when isoabienol occurs in a high amount. One may speculate that isoabienol can isomerize into *cis*-abienol. Sandaracopimaral, palustral and isopimaral were present in all analyzed EOs. However, these analytes were predominantly detected in twigs.

### 2.2. Geographical Origin

**Figure 4:**
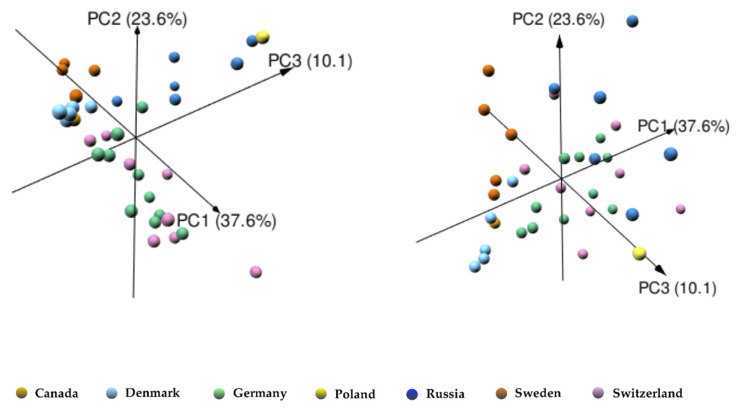
The 3D score plot of principal components PC1, PC2 and PC3 for EOs of PS based on the sesquiterpenes. Each sample is colored based on its origin.

Despite the similarity of the terpenoid profile among the investigated EOs of PS, a separation into the geographical origins was feasible. To visualize the differences in the collected EOs in terms of origin, a principal component analysis (PCA) on normalized data was performed. PCA is a well-known method for exploratory data analysis, which projects the original data onto a lower dimensional space of orthogonal components (principal components (PCs)), so that the first one explains the largest variance, the second one explains the largest variance, and so on [36,56,57]. In our case, the first three principal components (PC1, PC2 and PC3) explained 37.6%, 23.6% and 10.1% of the total variance, respectively, allowing the visualization of more than 70% of the information contained within the dataset in three dimensions (3D) (Figure 4). The corresponding loading plots are found in Supplementary Figure S4. Some of the EOs were well separated based on the first three PCs in terms of their origin, i.e., EOs from Denmark, Sweden and Russia. The EO of PS showed a great variability in their sesquiterpene content and pattern obtained from different geographical locations, whereas the Swiss and German EOs were not separated due to their closeness of collection locations. EOs from Russia were characterized by relatively higher values of oxygenated sesquiterpenes, while EOs from Switzerland and Germany exhibited higher value of sesquiterpenes hydrocarbons and the Danish EOs of guaia-6,9-diene. PCA verified that the geographical location influences the chemical composition of the second metabolites and has to be considered for the quality control which is in line with previous reports [37,38].

### 2.3. Classification Model

To discriminate between EOs of PS and closely related pine trees, EOs of PC, PMu and PN were analyzed by chromatographic and chemometric profiling. The chemical composition of these pine EOs were presented in Table 1 (**37**, **44**, **53**) and Supplementary Table S2. As shown in Supplementary Materials Figure S5, PCA was not able to discriminate between different congeners, nor after the elimination of the outliers **3**, **4** and **5**. Thus, a PLS-DA model was calibrated to distinguish five classes of pine EOs. The EOs **3**, **4**, and **5** were classified as chemotypes of PS characterized by a high amount of β-phellandrene and considered as one class (PS II). PLS-DA is a multivariate classification method, which is based on a PLS regression algorithm and aims to find linear combinations of the original variables (latent variables (LV)) that better separate each class [59,60]. In our study, before PLS-DA calculation, the fourth root for data preprocessing was applied, which had previously shown efficiency discriminating seized cannabis samples obtained by GC-MS using the fourth root [61]. After auto-scaling, the number of latent variables was optimized with three-fold cross validation, with five latent variables (LV) maximizing the Non-Error Rate (NER) [62]. The model was further validated with bootstrap and random resampling protocols. The obtained model resulted stable, with a NER ranging from 89% and 93% for bootstrap and random resampling, respectively (Table 2). The model sensitivity, which represents the percentage of correctly classified compounds for each class, was always greater than or equal to 90% with the exception of PMu, whose sensitivity values were nonetheless equal to or higher than 75% (Table 2) [62]. The comprehensive classification performances are presented in Tables S3 and S4.

**Figure 5:**
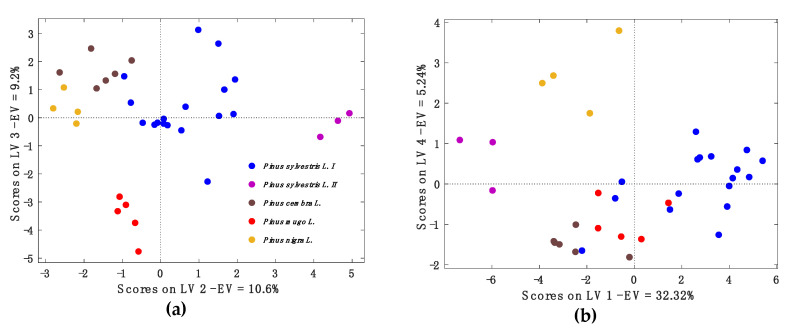
(**a**) The score plot of LV2 to LV3. (**b**) The score plot of LV1 to LV4.

As it can be seen from selected score plots (Figure 5), the EOs of PS I (blue) were clearly separated from PS II (violet), PC (brown), PMu (red) and PN (yellow) by the second and third latent variables (LV2 and LV3), whereas the score plot based on the first to the fourth latent variables (LV1 and LV4) additionally separated PC (brown) from PN (yellow). The corresponding loading plots are presented in Figure S6.

The PLS-DA model was able to separate PS (PS I/PS II) from the closely related pine EOs. This supports the existence of a proper primary EO of PS (even EOs of the chemotype PS II was separated). Furthermore, the developed model predicted the EOs of the test set in their corresponding taxonomic class (Table S5). The model can be used as a screening method to classify the EOs into their taxonomic specification. The classification of the EOs is crucial to ensure the quality and authenticity of the EOs and to avoid the possibility of confusion.

**Figure 6:**
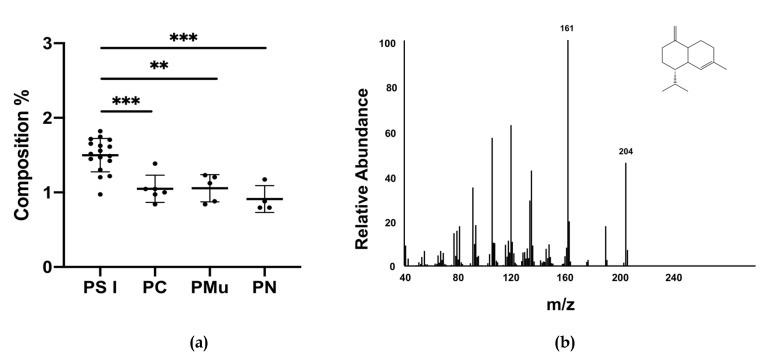
(**a**) γ-cadinene as potential chemical marker for characterizing primary EOs of PS I. Significance was tested using ANOVA followed by Tukey post-hoc test with *p* < 0.01: ** and *p* < 0.001: ***. (**b**) Mass spectrum of γ-cadinene.

To reinforce the statement of a proper primary EOs of PS I compounds with a regression coefficient > |0.05| for classification of PS I were considered (Figure S7). Among these compounds data of γ-cadinene were normally distributed and equal variances were assumed. The compound identified as γ-cadinene showed significant to highly significant difference of the mean value of PS I to closely related pine EOs and might serve as potential chemical marker for the classification of PS I (Figure 6). The result of the current study finally confirms suggestions made by our group after a preliminary study in 2016 (data not shown).

## 3. Materials and Methods

### 3.1. Chemicals and Solvents

C7–C30 saturated alkanes and heptane (puriss, p.a., Reag. Ph. Eur., ≥ 99%, *n*-heptane basis GC) were purchased from Sigma-Aldrich (St. Louis, MO, USA). Pure water was generated from an in-house water purification system from Labtec (Villmergen, Switzerland). Helium 6.0 and Hydrogen 5.0 were purchased from PanGas (Dagmersellen, Switzerland).

### 3.2. Primary EO of PS, PC, PMu and PN

Needles and twigs were obtained from PS (*n* = 36), PC (*n* = 7), PMu (*n* = 9) and PN (*n* = 6). A detailed overview of the used in-house codes, GPS coordinates and harvesting times can be found in the Supplementary Table S5. The EO of fresh cut (pieces of 1 cm) needles and twigs was obtained by industrial distillation. Subsequently, the EOs diluted in heptane and analyzed by GC-FID and GC-MS.

### 3.3. GC-FID Analysis for Chromatographic Fingerprint

GC-FID analysis was performed using a Thermo Fisher Scientific Trace Ultra gas chromatograph (Thermo Fisher Scientific, Waltham, Massachusetts, USA) equipped with a DB-wax capillary column (30 m × 0.25 mm i.d., film thickness 0.25 μm, Agilent, Santa Clara, USA). The temperature of the injection was 220 °C. The injection volume was 1 μL (autosampler AI3000, Thermo Fisher Scientific) using a split ratio of 1:50 with a split flow of 75 mL min^−1^. Helium was used as carrier gas at a constant flow rate of 1.5 mL min^−1^. The oven temperature was kept at 65 °C for 10 min and then heated to 220 °C with 5 °C min^−1^ and kept constant at 220 °C for 9 min. The temperature of the detector was 250 °C. The chromatographic profile was analyzed using the relative percentages of the individual components based on the FID response (peak area). The data were acquired with Chrom Card Trace Focus GC (Thermo Fisher scientific, version 2.9). Interlaboratory comparison was carried out with Systema Natura GmbH (Flintbek, Germany) using the same GC-FID method for the analysis for the chromatographic profile of randomly selected EOs (*n* = 8).

### 3.4. GC-MS Analysis for Chromatographic Profile

The chromatographic conditions from GC-FID were adopted to GC-MS analysis. The GC analysis was performed using a Thermo Fisher Scientific Trace Ultra gas chromatograph equipped with a BGB-wax capillary column (30 m × 0.25 mm i.d., film thickness 0.25 μm, Restek, Bellefonte, PA, USA) fitted with a guard column (1 m × 0.25 mm i.d, deactivated, Restek). The temperature of the PTV injection was 220 °C. The injection volume was 1 μl (TriPlus autosampler, Thermo Fisher scientific) using a split ratio of 1:50. Helium was used as carrier gas at a constant flow rate of 1.5 mL min^−1^. The oven temperature was kept at 65 °C for 10 min and then heated to 220 °C with 5 °C min^−1^ and kept constant at 220 °C for 9 min. The MS analysis was carried out on a Thermo DSQ II mass spectrometer detector operated in positive EI mode at 70 eV. Transfer line and ion source temperatures were set to 250 °C. Mass spectra were acquired in the full scan mode (mass range 40–300 *m*/*z*). Peak identification was performed using different libraries: *NIST* (version 2.2, 2014), *Adams* (fourth edition, 2007) and in-house libraries [58,63]. Retention indices (RI) were calculated according to the van den Dool and Kratz equation [64]. The used software was Thermo Xcalibur (Thermo Fisher scientific, version 2.2 SP1.48).

### 3.5. Statistical Analysis

PCA was performed with Rstudio (version 1.2.5019; packages: ggbiplot, version 0.55; pca3d, version 0.10). PLS-DA was performed by means of MATLAB (version R2019b) with a freely available classification toolbox [58]. The statistical analysis (Brown-Forsythe, ANOVA, Tukey post-hoc test) and illustrations were carried out using GraphPad Prism 8 (version 8.0.0 (224)) software.

PCA was performed on auto-scaled data of the chemical composition (sesquiterpenes) of primary EOs of PS (*n* = 36).

PLS-DA was performed on fourth root calculated and subsequent auto-scaled data. The dataset was composed of 35 EOs (PS I (*n* = 17), PS II (*n* = 3), PC (*n* = 6), PMu (*n* = 5) and PN (*n* = 4)) characterized by 39 compounds (Table S5). The threshold value for the separation of the classes was estimated using Bayes’ Theorem. Three-fold cross-validation was performed using venetian blinds splitting protocol and used to select the optimal number of latent variables based on Non-Error Rate [61]. Additional validation was performed using bootstrap and random resampling. The PLS-DA settings for all types of validations were reported in the Table S6. The developed PLS-DA model was applied to predict PS (*n* = 16), PC (*n* = 1), PMu (*n* = 4) and PN (*n* = 2) EOs (test set).

To determine potential chemical markers data (fourth root of chemical composition) were compared by using an ordinary one-way ANOVA followed by Tukey post-hoc test. Prior to ANOVA, normal distribution using Shapiro-Wilk test (α = 0.05) and homoscedasticity using Brown-Forsythe test (*p* < 0.05) were asserted.

## 4. Conclusions

A primary EO of PS was characterized by its chromatographic profile with a distinct sesquiterpene pattern followed by a diterpene area containing diterpenoids of labdane, pimarane or abietane type. Chemometric methods in combination with chromatographic profiling like PCA and PLS-DA were successfully applied to assign EOs of PS into their geographical origin and to differentiate closely related pine EOs. PLS-DA was established as a powerful screening tool in routine analysis and identification of EOs from PS.

## Figures and Tables

**Table 1:** RI values obtained from the literature (RI lit) [58] and calculated RI values (RI cal), spectral similarity index (RSI) and chemical composition (%, percentages of the total EO composition), of selected EOs (*n* = 24) of pine EOs. Further data are displayed in Supplementary Tables S1 and S2.

Compounds	RI lit	RI cal	RSI	1	2	3	4	5	6	7	8	9	10	11	12	13	14	15	16	17	18	19	20	21	37	44	53
**Monoterpene Hydrocarbons**																											
Tricyclene	1007	1013	932	-	0.6	-	-	-	1.1	0.4	0.9	0.6	0.4	0.4	0.9	0.8	1.2	0.2	0.4	3.1	0.8	0.4	0.5	0.4	-	0.7	0.2
α-Pinene	1026	1023	943	65.3	29.3	7.5	4.8	7.0	40.3	30.2	45.2	15.9	39.9	26.2	25.4	29.8	30.6	23.0	12.3	26.3	34.4	27.8	29.8	21.9	51.9	19.5	53.2
Camphene	1040	1065	959	1.3	3.3	0.6	-	-	6.3	1.4	4.0	2.3	1.8	1.6	3.8	3.6	5.0	1.1	1.9	1.4	4.2	2.0	2.8	2.1	0.8	2.6	1.2
β-Pinene	1114	1117	944	2.5	6.2	38.4	2.7	20.6	3.0	1.0	2.9	1.2	31.2	5.6	10.6	8.5	15.5	1.6	1.4	3.0	6.8	2.0	16.1	6.3	9.8	5.2	25.4
Sabinene	1110	1122	841	-	1.3	0.7	3.3	8.3	1.0	1.1	0.5	1.0	-	0.9	0.6	0.7	0.9	1.4	1.3	1.3	0.7	-	-	0.3	0.4	0.7	0.2
3-Carene	1135	1147	944	-	37.8	7.0	-	-	18.1	33.9	15.3	33.5	-	32.2	12.5	20.7	17.9	47.0	47.9	25.3	10.7	-	-	-	-	0.2	-
β-Myrcene	1155	1160	937	1.9	2.6	1.6	1.1	-	7.8	1.9	1.7	2.2	10.7	1.9	1.9	1.9	3.5	2.6	4.3	3.7	8.6	10.2	6.7	7.3	1.5	26.3	1.0
Limonene	1205	1202	908	14.3	0.7	2.7	12.6	2.1	0.8	0.5	0.7	4.7	3.4	0.5	1.9	0.5	0.6	0.6	0.5	7.7	6.5	13.9	7.8	6.7	2.4	14.9	3.3
β-Phellandrene	1183	1208	946	4.0	4.1	33.6	56.2	46.4	8.7	2.1	0.3	0.4	1.0	2.2	2.6	0.4	1.8	5.5	4.4	9.5	4.1	2.7	3.5	4.7	22.4	9.6	0.9
*p*-Cymene	1253	1265	885	-	1.5	-	-	-	2.0	0.3	0.4	2.7	0.8	1.2	0.8	0.9	0.4	0.9	0.9	0.2	0.1	0.5	0.3	0.3	-	0.5	0.4
Terpinolene	1271	1279	924	-	3.4	0.8	7.5	8.7	2.1	2.9	1.8	2.7	-	3.0	1.3	2.0	1.6	4.5	4.8	0.8	0.1	-	0.2	-	0.2	1.1	0.3
**Oxygenated Monoterpenes**																											
Bornyl acetate	1554	1571	941	-	-	-	-	-	3.4	0.3	0.5	0.3	-	0.3	0.3	1.9	4.4	0.3	0.6	1.3	3.9	3.5	2.8	2.7	0.2	0.9	0.6
α-Terpineol	1675	1674	875	-	-	-	-	-	-	-	0.5	0.7	-	0.6	0.2	-	-	0.5	0.2	0.3	0.3	0.3	0.3	0.4	-	0.3	0.1
**Sesquiterpene Hydrocarbons**																											
Longipinene	1539	1454	906	-	-	-	-	-	-	-	-	-	-	-	-	-	-	-	-	0.8	0.1	-	0.2	-	-	-	-
Copaene	1493	1479	944	-	-	-	-	-	-	-	0.3	0.5	-	0.3	0.3	0.2	-	-	0.2	-	0.2	0.3	0.1	0.2	-	-	0.2
Longifolene	1590	1548	914	-	-	-	-	-	-	-	0.3	-	-	-	-	-	-	-	-	0.3	0.2	-	-	-	-	-	-
β-Caryophyllene	1597	1585	950	-	0.6	-	-	-	0.7	5.7	4.8	2.8	2.0	2.3	3.3	2.1	1.7	1.3	1.2	1.8	0.9	1.9	0.9	0.8	0.3	3.0	1.9
Guaia-6,9-diene	-	1596	881	-	-	3.7	3.9	7.0	-	-	-	-	-	-	-	-	-	-	-	-	-	-	-	-	-	-	-
α-Humulene	1671	1653	917	-	-	-	-	-	-	0.9	0.8	0.5	0.3	0.5	0.6	0.3	0.4	0.3	0.2	0.5	0.4	0.3	0.2	0.3	0.3	0.6	0.4
γ-Muurulene	1692	1678	936	-	-	-	-	-	-	0.2	0.6	0.7	-	0.5	0.5	0.4	0.1	0.1	0.2	-	0.3	-	0.3	0.3	-	0.3	0.2
Germacrene d	1693	1697	945	-	0.6	-	-	-	-	2.2	3.4	2.7	1.4	1.7	2.3	1.2	1.1	0.3	0.4	0.3	0.7	1.2	1.8	1.8	6.8	4.0	8.9
β-Selinene	1717	1704	937	-	-	-	-	-	-	0.3	0.8	0.8	0.2	0.8	0.7	0.6	0.1	0.2	0.4	0.2	0.3	-	0.4	0.2	-	-	-
α-Selinene	1703	1710	925	-	-	-	-	-	-	0.4	0.7	0.7	-	0.8	0.6	0.6	0.2	0.3	0.3	0.3	0.3	-	0.3	0.2	-	0.6	-
α-Muurolene	1727	1715	944	-	0.8	-	-	-	-	0.5	0.6	1.1	0.3	0.9	1.1	0.7	0.3	0.2	0.4	0.3	0.6	0.8	0.7	0.6	-	-	-
Bicyclogermacrene	1735	1720	932	1.0	0.8	-	-	-	-	2.0	1.9	1.2	0.7	2.5	3.4	2.9	2.8	0.8	1.1	0.3	0.2	1.6	1.6	1.1	0.9	1.0	0.1
γ-Cadinene	1765	1749	903	2.1	1.9	-	3.0		0.9	4.7	6.8	12.2	2.9	7.5	10.2	8.6	2.9	1.5	3.4	2.0	2.2	8.7	5.3	4.1	1.0	2.1	0.6
**Oxygenated Sesquiterpenes**																											
Cubebol	1957	2040	924	-	-	-	-	-	-	-	0.3	0.7	-	0.4	0.6	0.5	0.1	0.2	1.2	0.6	0.7	1.3	0.8	0.9	-	-	-
Germacrene-d-4-ol	2018	2072	929	1.7	1.4	-	-	-	0.8	4.7	1.8	4.5	2.2	2.1	7.6	5.1	3.6	1.5	2.6	2.6	2.0	10.4	8.8	11.3	1.0	0.3	0.1
Spathulenol	2151	2114	916	-	0.3	-	-	-	-	-	-	-	-	-	0.2	0.3	0.2	0.3	0.6	1.1	1.4	0.4	0.9	1.1	-	-	-
τ-Cadinol	2169	2285	896	-	0.5	-	-	-	-	-	0.2	0.3	-	0.2	0.6	0.5	0.2	0.2	0.7	0.5	0.4	1.0	0.9	1.2	-	0.2	-
τ-Muurolol	2186	2177	926	-	0.6	-	-	-	0.4	0.3	0.2	0.4	-	0.2	0.7	0.6	0.3	0.2	0.7	0.5	0.5	1.0	0.1	1.1	-	0.3	-
α-Cadinol	2226	2222	925	1.1	1.5	-	-	-	1.2	0.6	0.3	0.6	0.3	0.3	1.4	1.2	0.9	0.4	1.2	1.2	1.0	2.0	1.8	2.6	0.2	0.7	0.1
Oplapanone	2545	-	955	-	-	-	-	-	-	-	-	-	-	-	-	-	-	-	-	0.3	0.9	-	0.2	0.9	-	-	-
**Oxygenated Diterpenes**																											
Manool oxide	2348	-	920	-	-	-	-	-	-	0.3	-	-	-	-	-	-	-	-	-	1.8	-	0.6	-	0.3	-	-	-
Isoabienol	2606	2727	-	4.8	-	-	-	-	0.9		-	-	-	-	-	-	-	-	-	0.5	0.6	-	-	12.6	-	-	0.1
Sandaracopimaral	2786	2744	905	-	-	-	-	-	-	-	-	-	-	-	-	-		0.4	0.6	-	-	0.3	0.1	0.2	-	-	-
*cis*-Abienol	-	-	-	-	-	-	-	-	-	-	-	-	-	-	-	-	-	-	-	-	-	-	-	0.2	-	-	-
Palustral	2850	2845	907	-	-	-	-	-	-	-	-	-	-	-	-	-	-	-	-	0.3	-	0.4	0.1	-	-	0.3	-
Isopimaral	-	2857	904	-	-	1.1	-	-	-	-	-	-	-	-	-	-	0.1	0.3	0.7	-	-	0.4	0.3	0.3	-	0.2	-

-: not detected.

**Table 2:** Classification parameters of the PLS-DA model in cross-validation, bootstrap and random resampling [61]. Sensitivity (Sn) and specificity (Sp) for each class, along with non-error rate (NER) and ratio of non-assigned compounds (n.a.) are reported.

Parameter	Class	Cross-Validation	Bootstrap	Random Resampling
Sn	PS I	0.94	0.91	0.93
	PS II	1.00	0.90	0.99
	PC	1.00	0.93	0.92
	PMu	0.75	0.75	0.80
	PN	1.00	0.95	0.98
Sp	PS I	0.94	0.91	0.92
	PS II	1.00	1.00	1.00
	PC	0.96	0.96	0.98
	PMu	1.00	0.97	0.98
	PN	1.00	1.00	1.00
NER	-	0.94	0.89	0.93
n.a.	-	0.06	0.17	0.10

## Supplementary Materials

The following are available online: Table S1: Chemical composition (%, percentages of the total EO composition) of the primary EOs of PS; Table S2: Chemical composition (%, percentages of the total EO composition) of closely related pine EOs; Table S3: Classification parameters of the PLS-DA model in fitting, cross-validation, bootstrap and random resampling. Sensitivity (Sn), specificity (Sp) and precision (P) for each class are reported; Table S4: Classification parameters of the PLS-DA model in fitting, cross-validation, bootstrap and random resampling. Sensitivity (Sn), specificity (Sp) and precision (P) for each class are reported; Table S5: Origin data of the primary EOs and their classification in PLS-DA analysis (EOs for PLS-DA development in bold, EOs for test set in italic); Table S6: PLS-DA settings of the used types of validations; Figure S1: Phylogeny of the genus *Pinus*. *Pinus* is divided into the subgenus *Pinus* and *Strobus.* Only the used species in this study are mentioned; Figure S2: Fragmentation pattern of the diterpenoids; Figure S3: The diterpenoid profile obtained from (a) needles and (b) twigs; Figure S4: The loading plot of principal components PC1 versus PC2 for EOs of PS based on the sesquiterpenes; Figure S5: (a) and (b) The score plot of PC1 and PC2 with and without outliers; Figure S6: (a) and (b) The loading plot of LV2 to LV3 and LV1 to LV4, respectively; Figure S7: Regression coefficients for the EOs of PS I.
