# Supplementary material for: Verification of Chromatographic Profile of Primary Essential Oil of Pinus sylvestris L. Combined with Chemometric Analysis
Source: Molecules. 2020 Jun 28;25(13):2973. doi: 10.3390/molecules25132973 (PMC7411901; doi:10.3390/molecules25132973)
Supplement: Supplementary file 1 [file molecules-25-02973-s001.pdf]

Supplementary Information

Verification of the Chromatographic Profile of Primary Essential Oil of *Pinus sylvestris* L. Combined with Chemometric Analysis

Martina Allenspach [1], Claudia Valder [2], Daniela Flamm [2], Francesca Grisoni [1] and Christian Steuer [1,*]

[1] Department of Chemistry and Applied Biosciences, Institute of Pharmaceutical Sciences, ETH Zürich, HCI, Vladimir-Prelog-Weg 4, 8093 Zürich, Switzerland

[2] Systema Natura GmbH, Konrad-Zuse-Ring 8, 24220 Flintbek, Germany, science@systemanatura.de

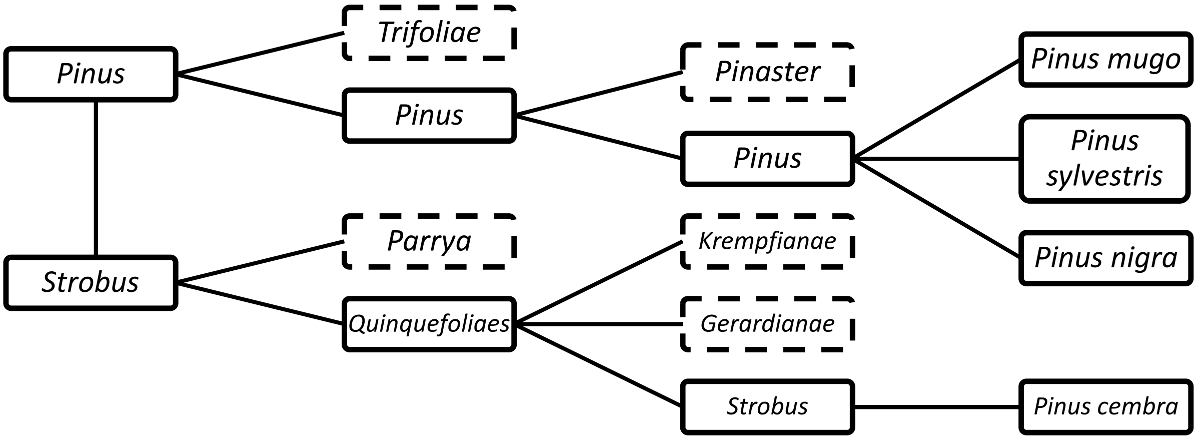

Figure S1. Phylogeny of the genus *Pinus*. *Pinus* is divided into the subgenus *Pinus* and *Strobus*. Only the used species in this study are mentioned. The information about the phylogeny is obtained from Gernandt, D. S.; Lopez, G. G.; Garcia, S. O.; Liston, A., Phylogeny and classification of Pinus. *Taxon* **2005,** *54* (1), 29-42.

Table S1. Chemical composition (%, percentages of the total EO composition) of the primary EOs of PS.

Compounds	22	23	24	25	26	27	28	29	30	31	35	36
Tricyclene	0.4	0.3	0.6	0.6	0.7	1.0	0.5	0.7	0.5	0.3	0.5	0.3
α-Pinene	38.8	20.0	38.1	58.9	23.6	27.0	11.2	40.7	19.9	26.4	16.1	14.0
Camphene	1.9	1.3	2.4	2.4	2.3	3.9	1.9	3.6	2.4	1.3	2.1	1.3
β-Pinene	7.5	2.5	8.9	1.6	2.0	1.4	1.1	23.7	13.2	1.6	5.7	1.6
Sabinene	0.4	0.4	0.4	0.1	0.9	0.6	1.1	0.2	0.5	1.4	1.0	1.3
3-Carene	-	-	12.4	-	30.1	19.0	43.2	-	17.6	28.9	22.1	34.9
β-Myrcene	3.6	1.5	3.5	8.1	8.5	5.9	2.4	9.9	6.4	4.3	-	-
Limonene	5.2	9.4	0.5	5.5	0.4	0.5	0.4	0.6	0.4	2.5	0.5	0.5
β-Phellandrene	5.3	9.4	0.4	0.3	0.5	0.4	1.3	0.8	0.6	1.0	5.9	4
p-Cymene	-	0.6	1.0	1.8	1.2	0.4	0.6	2.8	2.4	1.6	-	-
Terpinolene	-	0.2	0.9	0.4	2.6	1.8	3.3	0.2	1.8	2.2	2.1	3.3
Bornyl acetate	0.8	0.5	0.5	0.3	-	1.0	-	0.5	0.3	0.4	4.3	1.6
α-Terpineol	-	0.4	-	-	-	0.3	0.3	0.2	-	-	-	-
Longipinene	-	-	-	-	-	0.2	0.2	-	-	-	-	-
Copaene	0.2	0.3	0.2	-	0.2	0.4	0.4	0.2	0.3	-	-	-
Longifolene	0.7	0.8	-	-	-	0.3	-	0.3	-	0.4	0.4	-
β-Caryophyllene	1.0	1.2	0.8	2.4	7.5	6.5	3.1	1.1	4.4	6.6	1.2	1.6
Guaia-6,9-diene	-	-	-	-	-	-	-	-	-	-	-	0.8
α-Humulene	0.3	0.3	0.1	0.5	1.3	1.1	0.5	0.2	0.8	1.1	-	-
γ-Muurulene	0.4	0.6	0.4	0.2	0.4	1.2	0.7	0.4	0.6	-	-	-
Germacrene d	2.0	3.4	2.7	1.2	3.0	5.1	1.8	1.5	4.7	5.2	0.7	0.4
β-Selinene	0.4	0.5	0.4	0.1	0.5	1.4	0.8	0.4	0.9	-	-	-
α-Selinene	0.3	0.4	0.3	-	0.4	1.0	0.8	0.3	0.8	0.6	-	-
α-Muurolene	0.6	0.8	0.9	0.5	0.6	0.7	1.0	0.8	0.7	-	-	-
Bicyclogermacrene	0.4	1.5	1.7	1.3	1.8	3.2	1.5	0.8	3.9	2.0	-	-
γ-Cadinene	4.9	9.3	8.8	4.4	5.9	8.5	11.0	7.0	8.2	1.7	5.1	3.0
Cubebol	1.1	1.7	0.3	-	-	0.3	0.4		0.6	-	0.5	0.4
Germacrene-d-4-ol	9.0	18.7	3.4	2.1	1.2	0.5	1.3	0.7	4.4	2.5	12.3	5.4
Spathulenol	0.7	0.5	0.2	-	-	0.5	0.4	-	0.2	-	-	-
τ-Cadinol	0.7	1.5	1.0	0.5	0.3	0.3	0.9	0.3	0.4	-	0.8	0.5
τ-Muurolol	0.8	1.5	1.3	0.8	0.4	0.4	1.2	0.4	0.5	-	0.7	0.5
α-Cadinol	1.5	2.8	3.3	2.1	0.8	0.5	2.4	0.9	0.9	0.5	1.8	1.3
Oplapanone	1.9	-	-	-	-	-	-	-	-	-	-	-
Manool oxide	-	-	-	-	-	-	-	-	-	-	-	-
Isoabienol	3.9	2.1	-	3.3	1.2	-	-	-	-	4.7	0.7	13.4
Sandaracopimaral	-	0.5	-	-	-	-	-	-	-	-	0.3	0.5
cis-Abienol	-	-	-	-	-	-	-	-	-	-	-	1.0
Palustral	-	-	-	-	-	-	-	-	-	-	0.6	0.3
Isopimaral	-	0.9	-	-	-	-	-	-	-	-	2.6	1.7

-: not detected.

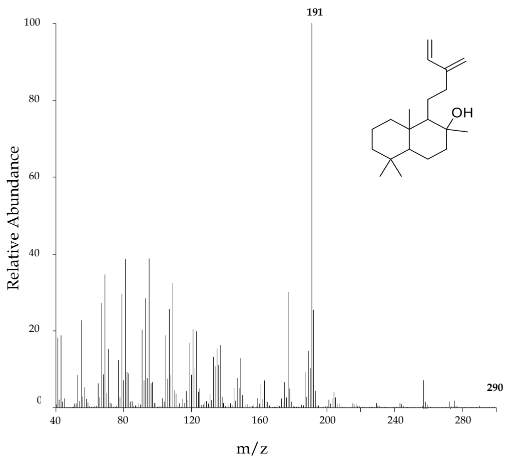

(a)

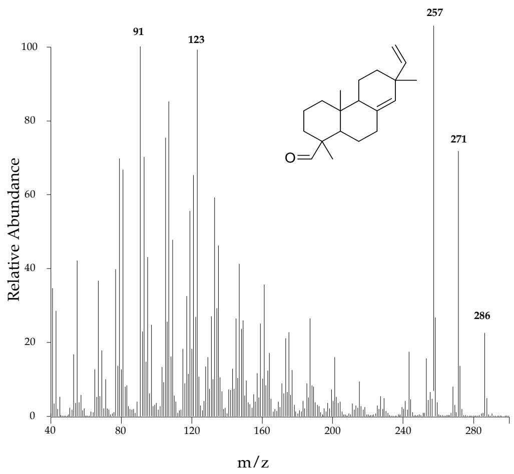

(b)

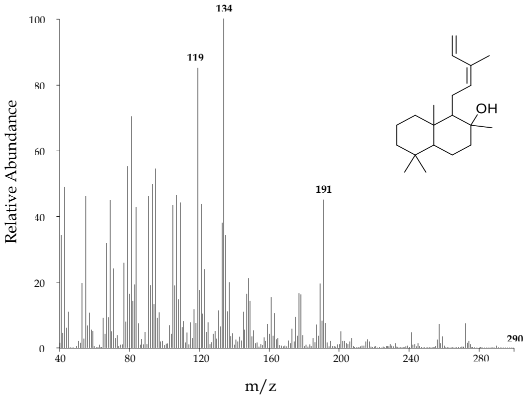

(c)

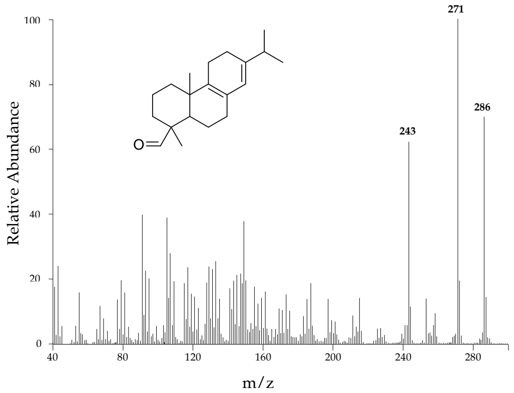

(d)

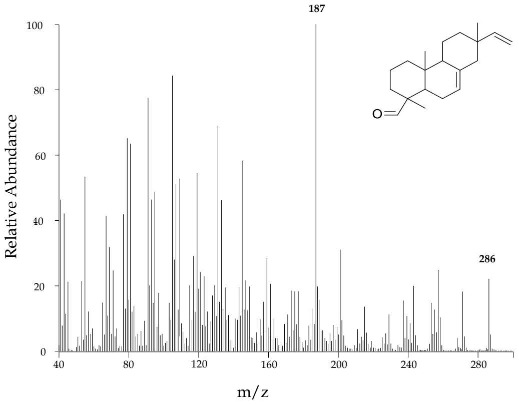

(e)

Figure S2. Fragmentation pattern of the diterpenoids: **(a)** isoabienol, **(b)** sandaracopimaral (SI:902, RSI:905), **(c)** *cis*-abienol (abienol: SI: 845, RSI 880), **(d)** palustral (SI:893, RSI:907) and **(e)** isopimaral (SI:868, RSI: 904).

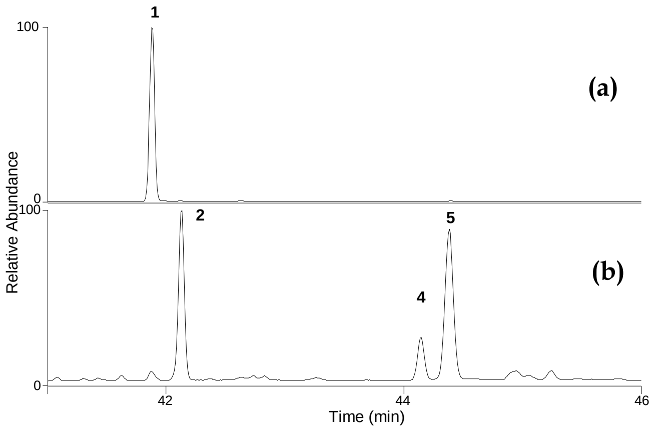

Figure S3. The diterpenoid profile obtained from **(a)** needles and **(b)** twigs with (1) isoabienol, (2) sandaracopimaral, (4) palustral and (5) isopimaral.

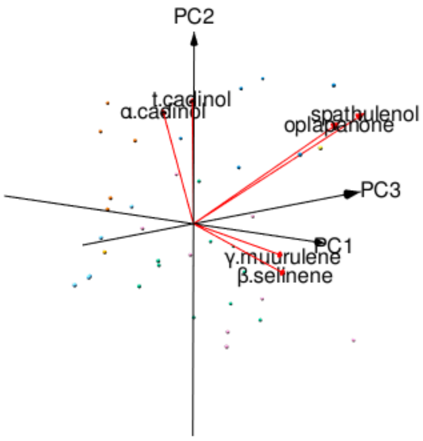

Figure S4. 3D loading plot of PC1, PC2 and PC3 for EOs of PS based on the sesquiterpenes.

Table S2. Chemical composition (%, percentages of the total EO composition) of closely related pine EOs.

**Compounds**	**38**	**39**	**40**	**41**	**42**	**43**	**45**	**46**	**47**	**48**	**49**	**50**	**51**	**52**	**54**	**55**	**56**	**57**	**58**
Tricyclene	0.1	0.1	-	0.2	0.2	0.1	1.1	0.5	0.9	2.5	0.4	0.3	0.9	1.6	-	-	-	0.5	-
α-Pinene	67.4	69.5	65.2	69.8	65.0	51.5	22.2	15.4	17.6	37.6	17.4	13.0	15.5	22.4	56.3	72.3	63.4	65.2	63.6
Camphene	0.6	1.2	0.7	1.7	0.9	0.8	4.3	2.3	3.0	11.6	1.4	1.0	3.6	5.5	1.1	1.5	1.1	2.0	1.2
β-Pinene	6.9	2.9	4.9	5.2	5.5	6.1	4.6	4.3	3.4	4.0	7.8	3.5	7.0	4.0	20.2	2.1	9.1	2.1	2.0
Sabinene	0.3	-	-	0.2	0.2	0.2	1.1	1.6	2.2	6.3	1.9	4.0	1.9	1.4	0.6	-	0.2	0.3	0.5
3-Carene	-	-	-	-	0.5	0.3	16.2	29.5	30.0	0.7	31.8	29.5	22.9	17.6	-	-	-	-	-
β-Myrcene	1.1	0.9	1.1	1.1	1.2	1.2	9.9	4.9	3.2	13.9	3.5	2.9	3.1	3.8	1.4	0.9	1.4	1.6	1.2
Limonene	3.1	7.5	9.2	3.1	6.2	6.1	4.9	7.8	2.2	0.5	4.7	6.5	1.2	3.5	2.3	2.0	2.6	2.4	2.4
β-Phellandrene	14.5	4.5	8.1	9.5	12.7	14.6	9.8	13.4	12.0	0.8	14.3	14.7	13.5	16.6	0.9	0.3	0.7	0.5	0.5
p-Cymene	-	-	-	-	-	-	0.8	0.5	1.4	0.8	0.3	0.4	0.8	-	1.2	0.3	0.8	1.6	1.2
Terpinolene	0.2	0.2	0.2	0.3	0.3	0.3	2.2	5.6	4.5	2.7	4.7	4.0	2.6	2.0	1.3	0.4	0.4	1.0	1.4
Bornyl acetate	-	0.5	-	0.8	0.2	-	6.0	3.2	3.0	13.9	0.9	0.7	6.5	10.0	0.4	0.4	0.2	1.2	0.5
α-Terpineol	-	-	-	-	-	-	0.3	2.1	0.4	-	1.1	1.6	0.6	0.5	-	0.4	-	0.1	0.5
Longipinene	-	-	-	-	-	-	-	-	-	-	-	-	-	-	-	-	-	-	-
Copaene	-	-	-	-	-	0.1	0.1	-	-	-	-	-	-	-	-	0.3	-	-	-
Longifolene	-	-	-	-	-	-	0.1	-	-	-	-	-	0.3	-	-	-	-	-	-
β-Caryophyllene	0.2	0.9	0.3	0.2	0.3	1.3	4.7	2.5	5.1	2.2	3.0	4.7	4.8	4.7	2.8	4.2	4.2	4.3	5.8
Guaia-6,9-diene	-	-	-	-	-	-	-	-	-	-	-	-	-	-	-	-	-	-	-
α-Humulene	0.3	0.5	0.4	0.3	-	0.6	0.8	0.4	0.8	-	0.5	0.8	0.9	0.7	0.4	0.8	0.6	0.8	1.0
γ-Muurulene	-	0.2	-	-	-	0.3	0.1	-	-	-	-	-	0.2	-	-	0.8	0.3	0.6	0.6
Germacrene d	3.5	8.5	6.1	4.7	2.1	4.7	1.6	0.7	2.2	0.5	0.7	0.8	3.8	0.5	10.7	8.0	15.4	12.7	15.5
β-Selinene	-	-	-	-	-	-	0.1	-	-	-	-	-	0.3	-	-	0.3	-	0.1	-
α-Selinene	-	-	-	-	-	-	-	-	-	-	-	0.4	0.3	-	-	-	-	-	-
α-Muurolene	-	-	-	0.1	0.2	0.4	0.5	-	0.5	-	-	0.4	0.2	0.3	-	0.2	-	0.1	-
Bicyclogermacrene	0.5	0.7	1.1	0.9	1.3	2.7	0.7	-	0.9	0.8	1.6	0.4	1.1	0.8	-	0.2	-	0.1	-
γ-Cadinene	0.5	1.2	1.2	0.9	1.7	3.7	1.6	0.6	2.3	0.5	0.4	2.0	0.9	1.2	0.4	1.9	0.4	1.2	1.1
Cubebol	-	-	-	-	-	0.2	-	-	-	-	-	-	-	-	-	-	-	-	-
Germacrene-d-4-ol	0.5	0.6	1.2	0.8	1.1	2.5	0.3	0.4	2.5	0.8	0.6	2.0	0.8	0.7	-	-	-	-	0.2
Spathulenol	-	-	-	-	-	-	-	-	-	-	-	-	0.1	-	-	-	-	-	-
τ-Cadinol	-	-	-	-	-	0.2	0.2	-	-	-	-	0.2	0.1	-	-	-	-	-	-
τ-Muurolol	-	-	-	-	0.1	0.3	0.3	0.4	-	-	-	0.3	0.1	-	-	-	-	-	-
α-Cadinol	0.1	0.1	0.2	0.2	0.3	0.6	0.6	0.9	0.7	-	0.3	0.9	0.3	0.4	-	-	-	-	-
Oplapanone	-	-	-	-	-	-	-	-	-	-	-	-	-	-	-	-	-	-	-
Manool oxide	-	-	-	-	-	-	-	-	-	-	-	-	-	-	-	-	-	-	-
Isoabienol	-	-	-	-	-	-	0.1	-	-	-	-	-	-	-	-	0.4	-	0.2	-
Sandaracopimaral	-	-	-	-	-	-	-	-	-	-	-	-	-	-	-	0.3	-	0.2	-
cis-Abienol	-	-	-	-	-	-	-	-	-	-	-	-	-	-	-	-	-	-	-
Palustral	-	-	-	-	-	-	0.3	0.6	-	-	-	0.2	0.8	0.5	-	-	-	-	-
Isopimaral	-	-	-	-	-	-	0.2	-	-	-	-	-	0.3	-	-	0.2	-	1.0	-

-: not detected.

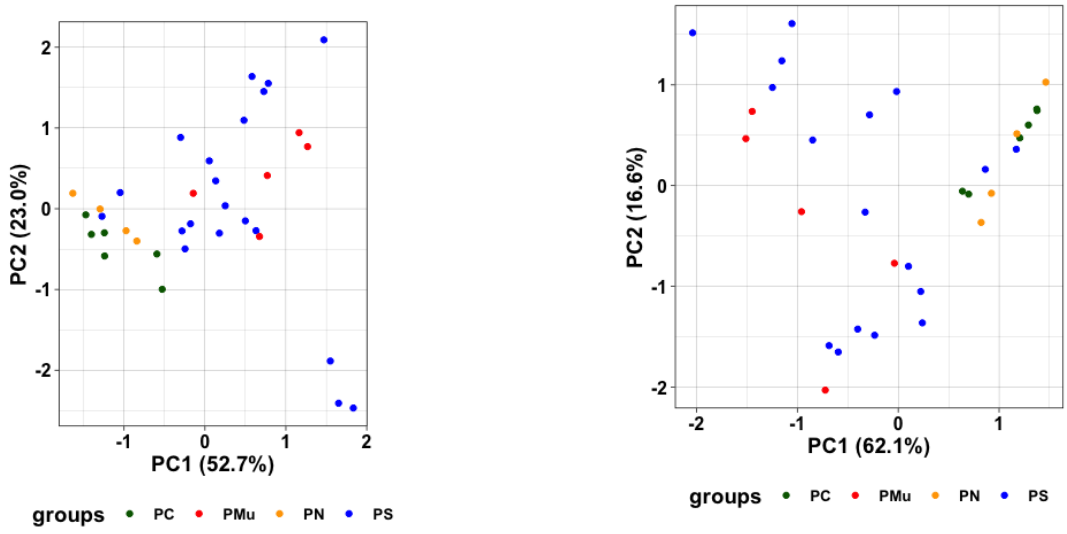

(a) (b)

Figure S5. (a) The score plot of PC1 to PC2 (without the predicted samples). The outliers **3**, **4** and **5** are located in the right lower hemisphere. **(b)** Score plot without the outliers **3**, **4** and **5** (PC: green, PMu: red, PN: yellow, PS: blue).

Table S3. Classification parameters of the PLS-DA model in fitting, cross-validation, bootstrap and random resampling. Error rate, along with non-error rate (NER) and ratio of non-assigned compounds (n.a.) are reported.

	Error Rate	NER	n.a.
Fitting	-	1.00	-
Cross validation (venetian)	0.06	0.94	0.06
Bootstrap	0.11	0.89	0.17
Random sampling	0.07	0.93	0.10

Table S4. Classification parameters of the PLS-DA model in fitting, cross-validation, bootstrap and random resampling. Sensitivity (Sn), specificity (Sp) and precision (P) for each class are reported.

	PS I	PS II	PC	PMu	PN
**Fitting**					
Sn	1.00	1.00	1.00	1.00	1.00
Sp	1.00	1.00	1.00	1.00	1.00
P	1.00	1.00	1.00	1.00	1.00
**Cross validation (venetian)**					
Sn	0.94	1.00	1.00	0.75	1.00
Sp	0.94	1.00	0.96	1.00	1.00
P	0.94	1.00	0.83	1.00	1.00
**Bootstrap**					
Sn	0.91	0.90	0.93	0.75	0.95
Sp	0.91	1.00	0.96	0.97	1.00
P	0.92	1.00	0.80	0.80	0.96
**Random resampling**					
Sn	0.93	0.99	0.92	0.80	0.98
Sp	0.92	1.00	0.98	0.98	1.00
P	0.91	1.00	0.92	0.86	0.99

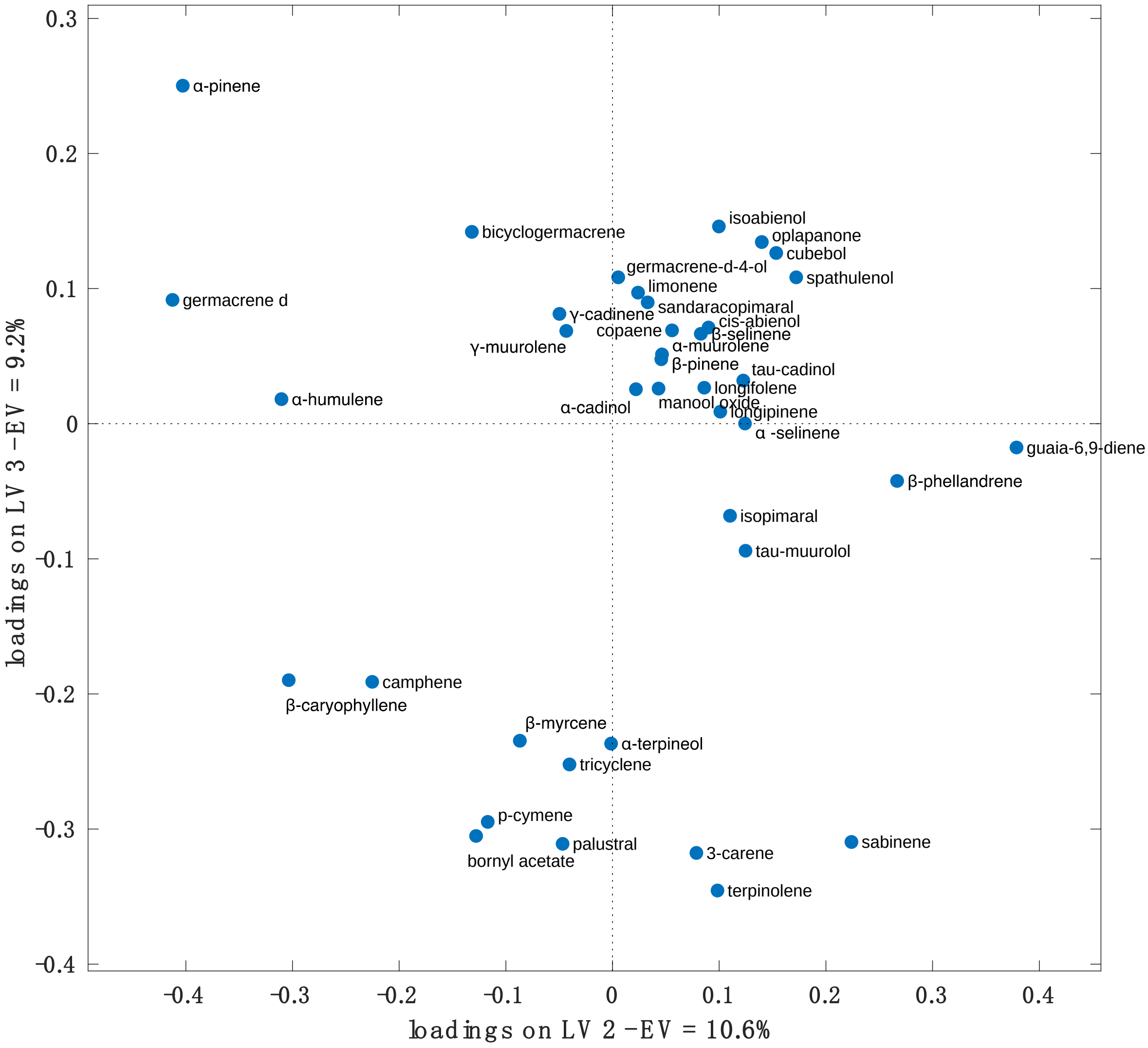

Figure S6. (a) The loading plot of LV2 to LV3.

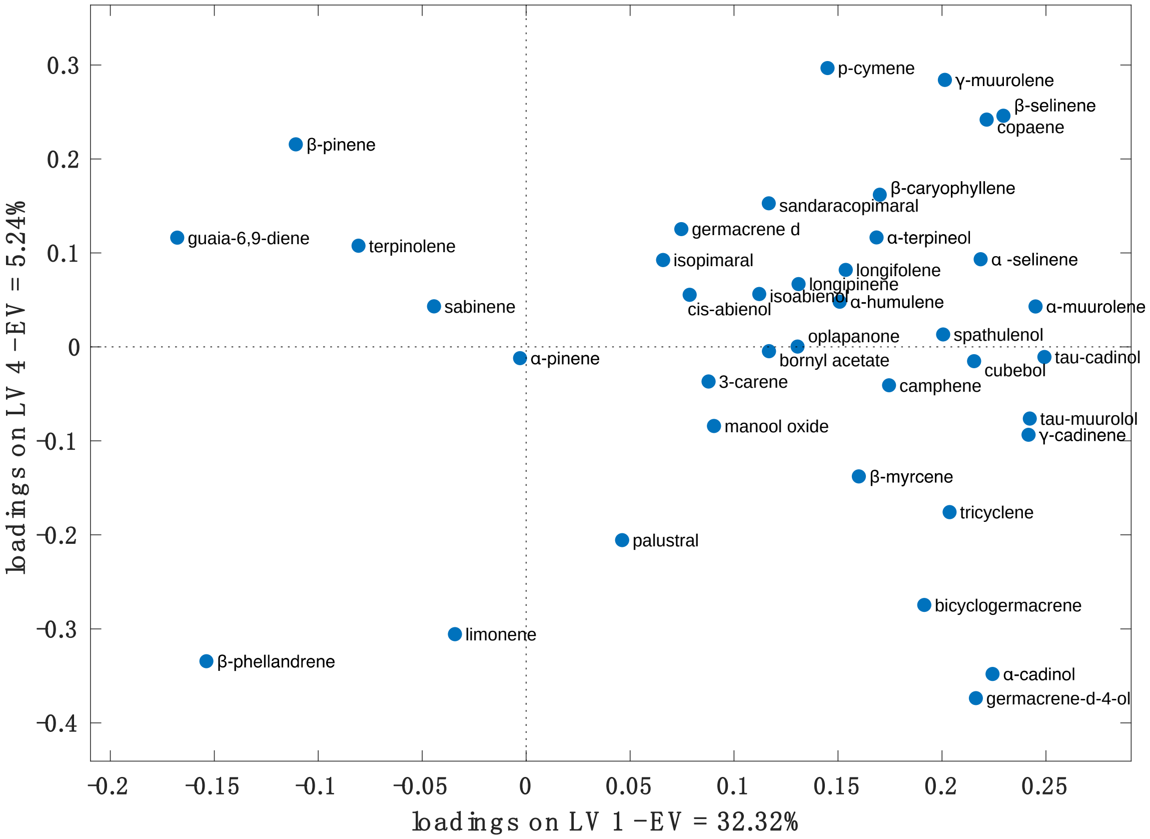

Figure S6. (b) The loading plot of LV1 to LV4.

Table S5. Origin data of the primary EOs and their classification in PLS-DA analysis (EOs for PLS-DA development in bold, EOs for test set in italic).

EO	Species	Country	GPS Coordinates	Harvesting Time	PLS-DA
1	*Pinus sylvestris* L.	Canada	-	-	**PS I**
2	*Pinus sylvestris* L.	Denmark	-	August 2019	*Predicted as PS I*
3	*Pinus sylvestris* L.	Denmark	-	August 2019	**PS II**
4	*Pinus sylvestris* L.	Denmark	-	August 2019	**PS II**
5	*Pinus sylvestris* L.	Denmark	-	August 2019	**PS II**
6	*Pinus sylvestris* L.	Denmark	-	August 2019	**PS I**
7	*Pinus sylvestris* L.	Germany	N47° 40′ 40″ E9° 10′ 23″	December 2019	**PS I**
8	*Pinus sylvestris* L.	Germany	N47° 40′ 40″ E9° 10′ 23″	December 2019	**PS I**
9	*Pinus sylvestris* L.	Germany	N47° 40′ 40″ E9° 10′ 23″	December 2019	*Predicted as PS I*
10	*Pinus sylvestris* L.	Germany	N47° 40′ 40″ E9° 10′ 23″	December 2019	**PS I**
11	*Pinus sylvestris* L.	Germany	N47° 40′ 40″ E9° 10′ 23″	December 2019	**PS I**
12	*Pinus sylvestris* L.	Germany	N48° 07′ 56″ E11° 34′ 21″	February 2020	*Predicted as PS I*
13	*Pinus sylvestris* L.	Germany	N48° 07′ 56″ E11° 34′ 21″	February 2020	*Predicted as PS I*
14	*Pinus sylvestris* L.	Germany	N48° 07′ 56″ E11° 34′ 21″	February 2020	*Predicted as PS I*
15	*Pinus sylvestris* L.	Germany	-	January 2020	*Predicted as PS I*
16	*Pinus sylvestris* L.	Germany	-	January 2020	*Predicted as PS I*
17	*Pinus sylvestris* L.	Poland	-	Winter 2018	*Predicted as PS I*
18	*Pinus sylvestris* L.	Russia	-	Winter 2018	**PS I**
19	*Pinus sylvestris* L.	Russia	-	Winter 2018	**PS I**
20	*Pinus sylvestris* L.	Russia	-	Winter 2018	**PS I**
21	*Pinus sylvestris* L.	Russia	-	Winter 2018	**PS I**
22	*Pinus sylvestris* L.	Russia	-	Winter 2018	**PS I**
23	*Pinus sylvestris* L.	Russia	-	Winter 2018	**PS I**
24	*Pinus sylvestris* L.	Switzerland	N47° 25′ 16″ E9° 16′ 31″	August 2019	*Predicted as PS I*
25	*Pinus sylvestris* L.	Switzerland	N47° 24′ 29″ E8° 30′ 24″	July 2019	**PS I**
26	*Pinus sylvestris* L.	Switzerland	N47° 24′ 29″ E8° 30′ 24″	August 2019	**PS I**
27	*Pinus sylvestris* L.	Switzerland	N47° 23′ 54″ E8° 32′ 36″	August 2019	**PS I**
28	*Pinus sylvestris* L.	Switzerland	N47° 23′ 54″ E8° 32′ 36″	August 2019	**PS I**
29	*Pinus sylvestris* L.	Switzerland	N47° 27′ 35″ E9° 31′ 28″	August 2019	**PS I**
30	*Pinus sylvestris* L.	Switzerland	N47° 24′ 29′ E8° 30′ 24″	February 2020	*Predicted as PS I*
31	*Pinus sylvestris* L.	Switzerland	N47° 24′ 29″ E8° 30′ 24″	February 2020	*Predicted as PS I*
32	*Pinus sylvestris* L.	Sweden	-	-	*Predicted as PS I*
33	*Pinus sylvestris* L.	Sweden	-	-	*Predicted as PS I*
34	*Pinus sylvestris* L.	Sweden	-	-	*Predicted as PS I*
35	*Pinus sylvestris* L.	Sweden	-	-	*Predicted as PS I*
36	*Pinus sylvestris* L.	Sweden	-	-	*Predicted as PS I*
37	*Pinus cembra* L.	Switzerland	N46° 27′ 35″ E9° 47′ 45″	November 2019	**PC**

38	*Pinus cembra* L.	Switzerland	N46° 27′ 35″<br>E9° 47′ 45″	November 2019	**PC**
39	*Pinus cembra* L.	Switzerland	N46° 27′ 35″<br>E9° 47′ 45″	November 2019	**PC**
40	*Pinus cembra* L.	Switzerland	N46° 27′ 35″<br>E9° 47′ 45″	November 2019	**PC**
41	*Pinus cembra* L.	Switzerland	N46° 29′ 23″<br>E9° 54′ 15″	November 2019	**PC**
42	*Pinus cembra* L.	Switzerland	N46° 25′ 45″<br>E9° 45′ 49″	November 2019	*Predicted as PC*
43	*Pinus cembra* L.	Switzerland	N46° 06′ 28″<br>E7° 55′ 38″	December 2019	**PC**
44	*Pinus mugo* TURRA	Switzerland	N47° 17′ 03″<br>E9° 24′ 39″	August 2019	**PMu**
45	*Pinus mugo* TURRA	Switzerland	N47° 17′ 03″<br>E9° 24′ 39″	August 2019	**PMu**
46	*Pinus mugo* TURRA	Denmark	-	August 2019	**PMu**
47	*Pinus mugo* TURRA	Switzerland	N46° 29′ 45″<br>E9° 50′ 19″	November 2019	**PMu**
48	*Pinus mugo* TURRA	Switzerland	N46° 29′ 45″<br>E9° 50′ 19″	November 2019	**PMu**
49	*Pinus mugo* TURRA	Germany	N47° 40′ 40″<br>E9° 10′ 23″	December 2019	*Predicted as PMu*
50	*Pinus mugo* TURRA	Switzerland	N47° 08′ 22″<br>E8° 32′ 09″	February 2020	*Predicted as PMu*
51	*Pinus mugo* TURRA	Switzerland	N47° 08′ 22″<br>E8° 32′ 09″	February 2020	*Predicted as PMu*
52	*Pinus mugo* TURRA	Switzerland	N47° 08′ 22″<br>E8° 32′ 09″	February 2020	*Predicted as PMu*
53	*Pinus nigra* J. F. ARNOLD	Switzerland	N47° 25′ 31″<br>E9° 15′ 32″	November 2019	**PN**
54	*Pinus nigra* J. F. ARNOLD	Switzerland	N47° 25′ 31″<br>E9° 15′ 32′	November 2019	**PN**
55	*Pinus nigra* J. F. ARNOLD	Switzerland	N47° 24′ 23″<br>E9° 20′ 25″	November 2019	**PN**
56	*Pinus nigra* J. F. ARNOLD	Germany	N49° 23′ 34″<br>E7° 3′ 38″	December 2019	**PN**
57	*Pinus nigra* J. F. ARNOLD	Switzerland	N47° 24′ 29″<br>E8° 30′ 24″<br>E8° 30′ 24″	February 2020	*Predicted as PN*
58	*Pinus nigra* J. F. ARNOLD	Switzerland	N47° 24′ 29″<br>E8° 30′ 24″	February 2020	*Predicted as PN*

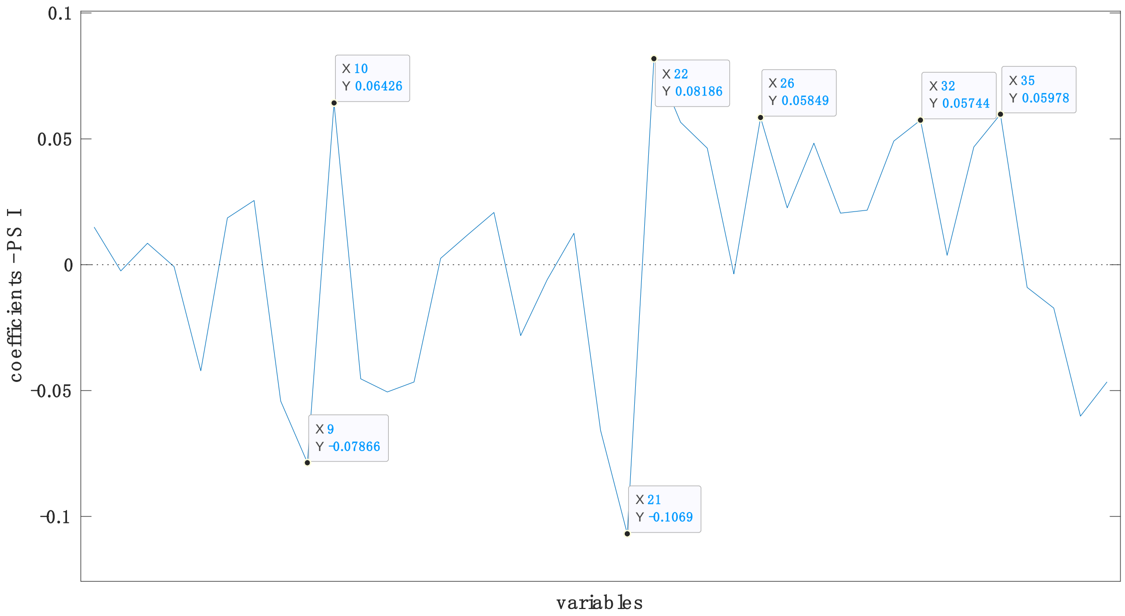

Figure S7. Regression coefficients for the EOs of PS I (9: β-phellandrene, 10: p-cymene, 21: germacrene d, 22 β-selinene: 26: γ-cadinene, 32: α-cadinol and 35: isoabienol).

Table S6. PLS-DA settings of the used types of validations.

	**Cross Validation**	**Bootstrap**	**Random Resampling (Montecarlo)**
Number of Latent Variables	5	5	5
Data scaling	4th root, autoscaling	4th root, autoscaling	4th root, autoscaling
Assignation criterion	Bayes	Bayes	Bayes
Validation	Venetian blinds	Bootstrap	Random resampling (montecarlo) of 20% of samples
Number of cross validation groups	3	100 (number of iterations)	1000 (number of iterations)
